# Expression of Ca^2+^-Binding Buffer Proteins in the Human and Mouse Retinal Neurons

**DOI:** 10.3390/ijms20092229

**Published:** 2019-05-07

**Authors:** Tamás Kovács-Öller, Gergely Szarka, Alma Ganczer, Ádám Tengölics, Boglárka Balogh, Béla Völgyi

**Affiliations:** 1 János Szentágothai Research Centre, University of Pécs, 7624 Pécs, Hungary; gergely.sz@gmail.com (G.S.); alma.ganczer@gmail.com (A.G.); tengo.adam2@gmail.com (Á.T.); boggancs2000@gmail.com (B.B.); volgyi01@gamma.ttk.pte.hu (B.V.); 2 Retinal Electrical Synapses Research Group, National Brain Research Program (NAP 2.0), Hungarian Academy of Sciences, 1051 Budapest, Hungary; 3 Department of Experimental Zoology and Neurobiology, University of Pécs, 7624 Pécs, Hungary; 4 Medical School, University of Pécs, 7624 Pécs, Hungary

**Keywords:** neuron, Ca^2+^, retina, parvalbumin, calretinin, calbindin

## Abstract

Ca^2+^-binding buffer proteins (CaBPs) are widely expressed by various neurons throughout the central nervous system (CNS), including the retina. While the expression of CaBPs by photoreceptors, retinal interneurons and the output ganglion cells in the mammalian retina has been extensively studied, a general description is still missing due to the differences between species, developmental expression patterns and study-to-study discrepancies. Furthermore, CaBPs are occasionally located in a compartment-specific manner and two or more CaBPs can be expressed by the same neuron, thereby sharing the labor of Ca^2+^ buffering in the intracellular milieu. This article reviews this topic by providing a framework on CaBP functional expression by neurons of the mammalian retina with an emphasis on human and mouse retinas and the three most abundant and extensively studied buffer proteins: parvalbumin, calretinin and calbindin.

## 1. Introduction

**Figure 1:**
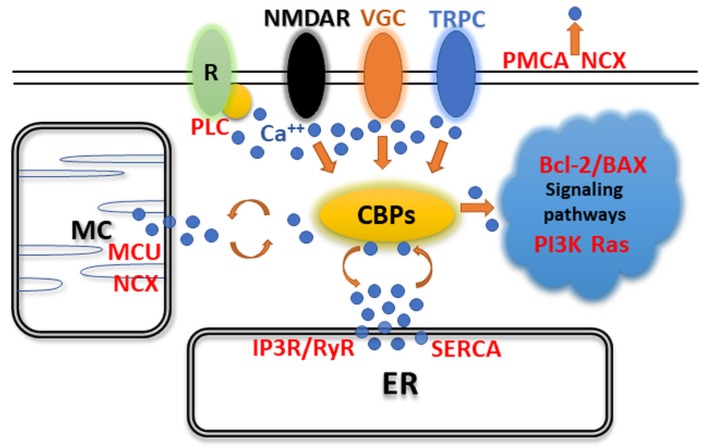
Signaling of EF-hand Ca^2+^-binding buffer proteins (CaBPs) shows a central role in Ca^2+^ maintenance. Effects of G-protein-coupled phospholipase C (PLC), N-methyl-D-aspartate receptors (NMDARs), voltage-gated calcium channels (VGCs), and transient receptor potential cation channels (TRPCs) can be buffered by CaBPs. Intracellular Ca^2+^-dependent signal transduction is significantly controlled by CaBPs. This Ca^2+^ originates from mitochondria (MC) and endoplasmic reticulum (ER). Mitochondrial calcium uniporter (MCU), inositol trisphosphate receptor, ryanodine receptor (IP3R/RyR), and sarco and endoplasmic reticulum Ca^2+^-ATPase (SERCA) control Ca^2+^ levels intracellularly, while the sodium–calcium exchanger (NCX) can also be found on the MC and cell membranes. Plasma membrane Ca^2+^-ATPase (PMCA) and NCX are responsible for the removal of Ca^2+^ from the cell.

Calcium ion (Ca^2+^) distributional changes have a central effect on living cells. As a second messenger, Ca^2+^ has a crucial role in intercellular communication and other neuronal functions. Therefore, intracellular Ca^2+^ levels must be maintained in a narrow and cell type-specific range. Besides the extrusion of Ca^2+^ into the extracellular space and sequestering in intracellular stores, living cells (including neurons) also utilize a set of Ca^2+^-binding buffer proteins (CaBPs) to adjust their actual Ca^2+^ concentration levels to the momentary functional requirement (Figure 1). The various CaBPs possess different Ca^2+^ affinities and kinetics, thereby affecting neuronal signaling dynamics differently. The characteristic functional effects of CaBPs therefore underlie the cell type-specific expression of these proteins, which ultimately provides a useful tool for researchers to utilize them as neurobiological markers for experimental studies.

Akin to other CNS loci, CaBPs have been shown to be widely expressed by neurons of the mammalian retina as well. In fact, there have been numerous approaches to reveal the identity of the diverse CaBP-expressing neuronal subtypes in retinas of various mammalian model species and the human tissue as well. CaBPs are divided into well-conserved protein families: annexins, pentraxins, C2-domain proteins, EF-hand proteins, vitamin-K-dependent proteins and the intraorganellar low-affinity, high-capacity Ca^2+^-binding proteins [1]. However, only a few members of the EF-hand family (parvalbumin—PV; calbindin—CaB; calretinin—CaR; the Ca^2+^ sensor calmodulin—CaM) and proteins of the S100 family are used as neuronal markers in the retina [2].

**Figure 2:**
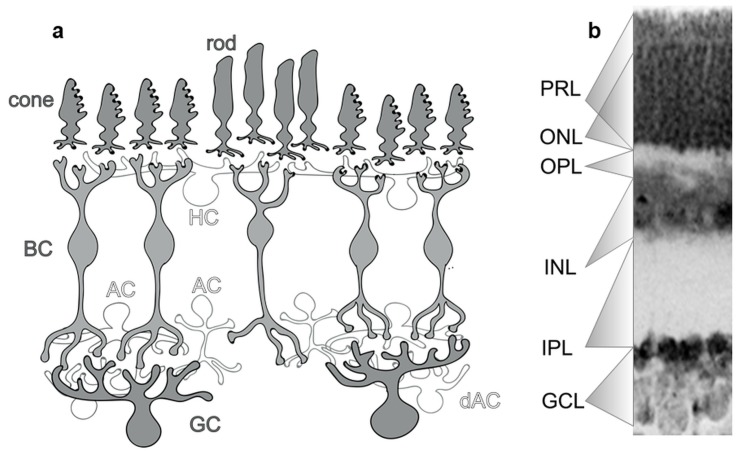
General structure of the mammalian retina. (**a**) The mammalian retina is a structurally complex multi-layered organ built from five major classes of neurons, namely, photoreceptors (PR), bipolar cells (BC) and ganglion cells (GC) that provide parallel pathways; and horizontal (HC) and amacrine cells (AC; and dAC for displaced amacrine cells) which are responsible for maintaining lateral inhibitory synapses in the outer and inner retina, respectively. Based on the localization of each cell type, the retina can be divided into well-defined anatomical layers, including the ganglion cell layer (GCL), the inner plexiform layer (IPL), the inner nuclear layer (INL), the outer plexiform layer (OPL), the outer nuclear layer (ONL) and the photoreceptor layer (PRL). (**b**) These layers are visible on a fluorescent Nissl-stained mouse retinal optical section (whole thickness: 200 µm).

The mammalian retina is a well-organized, layered structure that serves the first steps of visual perception, including the phototransduction as well as initial analysis of information and transmits this towards the brain. It is divided into ten layers with five neuron classes including retinal ganglion cells (rGCs), amacrine cells (ACs), bipolar cells (BCs), horizontal cells (HCs) and photoreceptors (PRs) (Figure 2). These cell types can be further separated into numerous morphological subtypes, most of which express at least one or more CaBPs. CaBP expression is rarely restricted to a single cell type in the retina but rather CaBPs are expressed by several neuron subtypes in each of the cell classes. Moreover, it is easier to find interspecies differences in CaBP labels than consistencies. Among the above mentioned CaBPs, we will focus this review on PV, CaR and CaB expression as the most prominently expressed proteins in retinal neurons and will mention other locally important CaBPs (Calcium-binding Proteins: calmodulin—CaM, recoverin—REC, secretagogin—SCGN, etc.) occasionally.

Out of all the CaBPs, CaM is the most ubiquitous, found in all eukaryotic organisms including yeasts. It has a central role in regulating cell function and protein expression including CaMKII, which has been described (among many other functions) as an important regulator of gap junction conductance [3,4]. The expression of CaM is very homogenous among vertebrates. Although CaM might play a role in phototransduction in the *Drosophila* [5], it could not be detected in PRs of vertebrates, questioning its direct role in phototransduction in their *Drosophila* counterparts [6].

CaB is a 28-kDa globular-shaped protein that contains six EF-hand (helix-loop-helix motif) domains. Four of them form the two active Ca^2+^-binding sites and two, further modified, domains lost their Ca^2+^-binding capability [7]. Importantly, CaB has a role in buffering the Ca^2+^ inflow induced by glutamate receptor stimulation and in Huntington disease [8,9,10]. CaR, as the name suggests, is abundantly expressed in the retina, labeling numerous cells. Previous results have shown CaR expression within the retinorecipient SCN layers as well [11]. Five out of its six EF-hand domains are functional and partake in Ca^2+^ buffering [1]. PV is a small (~12 kDa) EF-hand Ca^2+^ buffer widely expressed in the retina and other tissues as well, with two functional Ca^2+^-binding sites [1]. From the retina’s point of view, PV is perhaps the most interesting among all the CaBPs, since it labels mostly GCs. S100B and REC are both small, CaM-like EF-hand CaBPs that have a rather subtype-specific expression pattern among retinal neurons. S100B is present in all retinal layers, mostly in glial cells. In addition, S100B also serves as a Ca^2+^ sensor in cone photoreceptors (but not in rods) of the murine retina at the PR-BC synapses [12,13]. REC inhibits rhodopsin kinase thus it has been mainly found in PRs where it plays a role in the photo-recovery phase of the visual cascade [14]. Even though CaBP expression of retinal neurons in various mammals will be summarized, our goal is to focus this review on expressional patters in the human retina and compare it to expressions in the most popular animal model, the mouse.

## 2. Expression of CaBPs May Alter Neuronal Activity in the CNS

The molecules serving as chelators for Ca^2+^ contain negatively charged groups arranged into a specific geometry that enhance chemical coordination of Ca^2+^. Proteins with appropriately spaced acidic side-chain residues (e.g., glutamate, aspartate) and/or backbone carbonyl groups provide the ‘cage’ in which a Ca^2+^ fits in. Different types of CaBPs (annexins, C2-domain proteins, EF-hand proteins, pentraxins, vitamin-K-dependent proteins, and intraorganellar low-affinity, high-capacity Ca^2+^-binding proteins) [1,15] differ in the way they bind Ca^2+^. In general, the term ‘Ca^2+^ buffer’ is applied mostly to a small subset of cytosolic proteins of the EF-hand family, including PV and isoforms, CaB-D9k (9 kDa), CaB-D28k (28 kDa) and CaR. Some EF-hand proteins, such as CaM and S100, are ‘Ca^2+^ sensors’ that upon Ca^2+^ binding, change conformation and interact with specific targets [2]. However, when they are present in sufficiently high concentrations, Ca^2+^ sensors also function as Ca^2+^ buffers.

Members of the EF-hand family show a structural diversity in their EF-hand motifs [16]. These structural differences determine Ca^2+^ affinities and kinetics (binding and dissociation speeds) of CaBPs, which in turn modulate the spatiotemporal aspects of cytosolic Ca^2+^ signals and spiking activity of neurons. For example, due to the delayed Ca^2+^ binding of PV, the rising phase of Ca^2+^ concentration increase does not change (first 100 ms). On the other hand, the initial phase of the Ca^2+^ concentration decay is enhanced as PV finally takes effect, thus the intracellular Ca^2+^ wave becomes more transient. In addition, during the later decay phase of a signal, Ca^2+^ start dissociating from PV, enhancing neuronal activity. At the same time, CaB and CaR bind Ca^2+^ faster (even during the Ca^2+^ concentration rising phase), thus truncating stimulus-elicited Ca^2+^ responses (and spiking frequency) throughout the depolarization [1]. In addition, Ca^2+^ buffering by CaB also delays the Ca^2+^-dependent inactivation of voltage-dependent Ca^2+^ currents [1,17].

In summary, the actions of various CaBPs greatly affect neuronal excitability, activity patterns [18,19] and functions, such as short-term plasticity or LTP [17,20,21,22,23,24]. GABAergic inhibitory interneurons throughout the CNS for example often display PV expression and besides the presence of certain voltage-gated ion channels (e.g., Kv3.1; [25,26,27,28]), their spiking characteristics are strongly affected by the presence of PV [29,30]. Contrary to the extensive CaBP expression by mammalian retinal neurons, it is largely unknown if similar effects on neuronal activity are exerted as well. In coming sections of this review, however, we will summarize published data regarding this issue and speculate on possible CaBP function in neuronal activity.

## 3. CaBP Expression in PRs

REC is the only neuronal CaBP that is expressed in all PR cells in all examined mammalian species [31], including humans [32,33,34,35] and mice [36]. REC inhibits rhodopsin kinase and thereby reduces the phosphorylation of rhodopsin. Upon light stimulation, PRs hyperpolarize, the Ca^2+^ concentration drops and inhibition by REC is decreased. In turn, rhodopsin kinase is released from inhibition and can phosphorylate metarhodopsin II (activated form of rhodopsin) that leads to a more rapid inactivation and faster kinetics [14]. The Ca^2+^-bound form of REC in dark slows down the activity of rhodopsin kinase, thereby prolonging the light sensitivity of rhodopsin. Interestingly, REC is located in the cytosol when the intracellular Ca^2+^ level is low, and the ion is not bound to the protein. On the other hand, upon Ca^2+^ binding, a conformation change reveals the hidden myristoyl group that can now anchor to the disc membrane [37].

**Figure 3:**
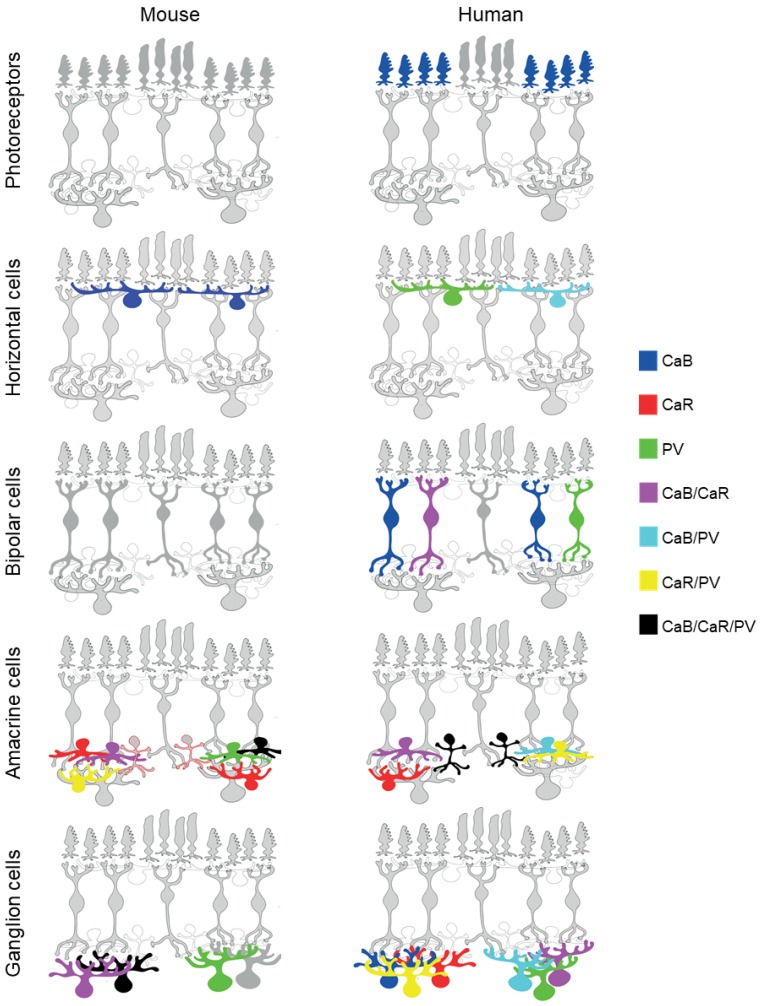
Parvalbumin (PV), calretinin (CaR) and calbindin (CaB) expression of mouse and human retinal neurons. Photoreceptors show little to no reactivity to the three examined CaBPs; in the PRL of the murine retina, no staining for CaB, CaR or PV has been demonstrated. At the same time, human cones (S, L/M) can all be labelled using CaB. CaB is expressed by HCs in both murine and human retinas. Although PV reactivity in mouse HCs has also been reported in one particular study, its presence remains questionable as no further experiments have succeeded in reproducing these results. On the other hand, the human retina decidedly contains a HC subtype that is labelled by both CaB and PV and one subtype that solely expresses PV. While BCs of the mouse retina show no reactivity to either CaB, CaR or PV, certain cone BC subtypes of the human retina can be reliably labelled by CaB, with one distinct population showing CaB+/CaR+ and PV+ immunoreactivity. The large AC population can be divided into many subtypes whose CaBP expression can differ considerably. However, CaR seems to be a reliable label for ACs in general, with CaB and PV expressed by certain (often undefined) cell populations as well. In the mouse retina, CaR+, CaR+/CaB+, CaR+/PV+ and CaR+/CaB+/PV+ AC populations exist. AII ACs (marked with a red outline), however, pose an interesting question as they are the only AC subtype in the mouse retina that express CaR according to labels in the CaR-GFP mouse retina but show no visible reaction to an a-CaR immunocytochemistry. AII ACs of the human retina, on the other hand, are decidedly CaR+/CaB+/PV+, while other subtypes demonstrate CaR+/CaB+, CaR+/PV+ and CaB+/PV+ labels. Like ACs, GCs of both the mouse and human retina also exhibit a considerable variety in their CaBP labels. Out of the two species, murine GCs appear to display fewer combinations of CaBP reactivity, while almost all potential combinations are represented throughout the many different populations of human GCs.

Besides REC, other CaBPs seem to be expressed more selectively. Cones have been successfully detected with antibodies that recognize CaB in various mammalian species, including humans and other primates [33,34,35,38,39,40,41,42,43,44]. Both S and L/M cones express CaB in the human retina [34]. However, CaB is not expressed in mouse (Figure 3) [36], rabbit [39] and rat retinas [45]. Interestingly, PRs of all examined species are devoid of both PV and CaR, while, at the same time, fetal monkey and human cones can temporarily be labeled by CaR [43,46], although its expression becomes completely downregulated after birth (Figure 3). The function of CaB in cones is not known but the lack of it in some species strongly suggests that it is not a crucial element of the phototransduction cascade. Interestingly, PRs also show no CaM immunoreactivity in the vertebrate retina [6], even though CaM is ubiquitous and mediates many crucial processes in eukaryotic cells.

## 4. CaBP Expression in HCs

REC HCs receive their input from PRs and provide lateral feed-back inhibition to PRs and/or feed-forward inhibition to BCs [47,48,49]. This spatially offset inhibition forms the backbone of the inhibitory surround receptive field for neurons in the retina. Two main HC populations exist in the mammalian retina: axon-bearing (B-type) and axonless (A-type) (Table 1). The latter one has a wider, more symmetrical radiating dendritic field and a large, robust soma and they maintain contact with both rods and cones through their dendrites. Contrary, axon-bearing cell inputs are segregated between dendrites and the axon terminal [50,51,52]. The general pattern is that the axon-bearing type is universal, while the presence of axonless HCs is species dependent [53,54,55]. While a single subtype of axon-bearing HC is present in the murine retina [56,57,58,59,60], the human retina maintains three distinct B-type HCs (HI, HII, HIII) and no A-type cells [61,62,63,64,65].

HCs in most mammalian species express CaBPs [6,36,44,45,56,66,67]. Both the human and the mouse outer retinas, including HCs, are devoid of CaR [36,44,45] (Figure 3). One of the earliest publications in the topic reported PV expression in mouse HCs [56]. However, later publications failed to demonstrate similar PV expression in mouse HCs via both immunocytochemistry [36] (our own unpublished observations) and genetical PV-tagging in PV-GFP mice [68] (our own unpublished observations). This discrepancy in related descriptions might be due to nonspecific labeling of available antibodies in the early 1990s or PV expressional changes in the mouse retina. In fact, PV expression has been shown to display a circadian rhythmicity and can be induced by pathological insults in the rat retina (for more details, see Section 8 of this review). Contrary to this inconsistency regarding PV, all descriptions agree on the CaB expression of mouse HCs [36,45,56,69]. In fact, CaB immunolabels completely disappear in the CaB null mutant mouse [66]. In the human retina, PV is expressed in HI and HII HCs, whereas CaB is solely expressed by HII HCs (Table 1) [36,44]. HII cells therefore appear as CaB/PV-dual-labeled cells with some compartmentalization of the two proteins (Figure 3): CaB is present in the somata and finer (likely axonal) fibers and PV is mostly found in thicker dendrites [44].

## 5. CaBP Expression in BCs

There have been 11–14 morphologically distinct BC subtypes identified in a wide range of mammalian retinas, including the mouse and the human retinas [32,34,35,44,65]. One of these populations has been identified as the rod BC that receives inputs solely from rod PRs. This cell type is well described in most examined species as it is specifically expressing the α-subunit of the enzyme protein kinase C (PKCα). The remaining BC subtypes are either ON- or OFF-cone BCs (5–6 subtypes in each), some of which express specific markers that can be utilized for histological targeting. CaBPs are among these markers, thus specific antisera have been widely utilized to identify mammalian BCs. In the primate retina, including humans, three diffuse BCs (DB1, DB2, DB3) and one flat midget BC (FMB; forming flat contact with cones) comprises the OFF- population, whereas three diffuse BCs (DB4, DB5, DB6), the invaginating midget BC (IMB; forming invagination synapse with cones) and a blue cone-specific BC (BB) has been characterized morphologically as ON-cone BCs [65].

At least four out of these eight human cone BC populations can be stained with sera recognizing CaBPs. REC is unique amongst CaBPs as it is specifically expressed (besides PRs) by FMB cells [32,34,35]. While the mouse retina lacks the midget system, the type 2 cone BC subpopulation of OFF BCs expresses REC in a similar fashion to FMBs in humans and other primates. Moreover, just like in the human retina (and other primates), REC is selective only for this subtype, providing the unique opportunity for selective targeting and studying this cell in the mouse retina as well. While this specific labeling of a single BC type with a-REC sera seems widespread among mammals (mouse [42], ground squirrel [70], primate [33,71]), some retinas contain an additional ON REC-positive cone BC subtype as well (rat [72,73]; rabbit [39]). At the same time, the strongly nocturnal microbat seems to be an exception from the unique staining hypothesis as REC positivity was found in multiple cone BCs including both OFF- (type1, type2 and type3) and ON subtypes (type4, type6 and type7). Interestingly, the sole primate retina that displayed two sets of REC-expressing BC subtypes (1 ON and 1 OFF) so far is the grey mouse lemur (*Microcebus murinus*), which is also a nocturnal animal [33]. Whether the nocturnal lifestyle requires REC expression in more BC populations than in diurnal retinas is yet unknown, mainly due to a lack of functional description for REC in BCs. REC is a neuronal CaBP that inhibits rhodopsin kinase in PRs and thereby reduces the phosphorylation of rhodopsin (see above). However, it is less known what role REC may play in BCs of the mammalian retina. As a CaBP, it may interfere with Ca^2+^-dependent mechanisms of expressing BCs such as neurotransmitter release. Alternatively, REC may regulate the activity of kinases in a similar fashion as REC inhibits rhodopsin kinase in rods.

Contrary to REC, CaB is expressed in at least three cone BC subpopulations in the human retina: in one OFF cell (DB3; [42]; [34,35,44]); and two ON BCs (likely DB5 and DB6) (Figure 3, Table 1) [34,35,42,44]. A similar expression pattern of CaB, by OFF DB3 and ON DB5 cone BCs, has been reported in all examined primates [33,74,75,76]. On the other hand, BCs in the murine retina show no CaB expression [36,77]. Whether this distinction between mouse and primate retinas is the consequence of different lifestyles (nocturnal vs. diurnal) or some other aspects is not clear yet. However, similar to the mouse, the nocturnal rat retina has no CaB+ BCs either [78,79], while other diurnal retinas, including the rabbit, contain CaB-expressing BCs [39,80]. The CaB+ ON BC of the rabbit retina maintains a narrowly stratifying axonal terminal in stratum 4, close to the rod BC terminals, thus resembling the primate DB6 cells that also express CaB. The similar morphological appearance and biochemistry of the two neuron populations may suggest a similar function that they play for vision.

CaR and PV, similar to CaB, are not expressed by murine BCs and are largely absent from BCs in retinas of the other mammalian species as well. There is one description on CaR/CaB dual expression in one set of human BCs that shows morphological similarities with IMB or DB4 cells [34,35,44]. The human retina also contains a BC population that expresses PV and maintains large axonal fields with axons branching mostly in the OFF-sublamina with some intrusions in the ON layer too [35], features that identify these cells as the giant bistratified BCs [54]. Therefore, apart from these two latter examples, one can conclude that CaR and PV are preferentially expressed in inner retinal neurons in most mammals, including humans (Figure 3). One more discrepancy between the CaBP expression of human and mouse retinas is represented by the expression of a recently cloned EF-hand protein named SCGN. This protein is expressed in several subpopulations of cone BCs in the mouse retina ([81]; types 2, 3, 4, 5, 6 and 8) but only expressed by ACs in humans [44]. Moreover, several BC populations of the rat and rabbit retinas also appear to be SCGN-positive. While there are interspecies differences, the SCGN-positive BCs in the rat and rabbit appeared to be similar to those of the mouse. This distinction, however, is not a primate vs. non-primate difference in SCGN expression as the macaque retina has been shown to express SCGN in DB1 BCs and not solely in ACs like in the human retina [82].

As discussed above (Section 1) CaB is considered to be a quick buffer protein and has been reported to buffer during the first 100 ms of Ca^2+^ transient rising phases to serve as a Ca^2+^ source for later phases, decreasing the effects of the Ca^2+^-dependent inactivation of voltage-dependent Ca^2+^ currents, thereby prolonging the response decay [1,83,84,85,86]. Because of that, one may expect CaB-expressing BCs to display a rather sustained response to the onset (like primate DB5 and DB6) or the offset (primate DB3) of light stimuli. Unfortunately, there is no information available about the light-evoked activity of the various BC subtypes in the primate retina to support this hypothesis. Moreover, mouse BCs display a variety of transient and sustained light responses (either with OFF or ON polarity) despite the lack of any CaBP expression. This indicates that while CaBPs may modulate, they do not determine the transiency of BC light responses.

Besides members of the classical EF-hand Ca^2+^ buffer family, Haeseleer and colleagues [87] identified five additional Ca^2+^-binding buffer proteins with high homology to calmodulin (CaBP1, CaBP2, CaBP3, CaBP4 and CaBP5) that are expressed in the mammalian retina. CaBP1 and CaBP5 has been shown in different subsets of BCs. CaB5-expressing cells in the human and macaque retinas have been identified as OFF DB3 and ON DB4 BCs, whereas CaBP+ cells in the mouse showed morphological similarities to the type3 (OFF) and type5 (ON) BCs [42]. Finally, mouse type4 cone BCs express calsenilin, another less known CaBP as a marker [88]. However, similar labelling in other species was negative or inconclusive for BCs.

There is also insufficient information about the signaling pathways that are initiated in the retina by the various CaBP-expressing BCs, the DB6 BC being the only documented exception (Table 1). The CaB+ DB6 BCs of the marmoset retina have been shown to establish a signaling pathway by providing synapses to narrow thorny GCs that project specifically to the K1 koniocellular layer in the LGN. This pathway is sensitive to rapid movement and through projections from LGN K1 to association in visual areas, it contributes to residual visual functions (‘blindsight’) as well [89].

## 6. CaBP Expression in ACs

As the inhibitory neurons of the inner retina, ACs contribute to vision by modulating the activity of inner retinal pathways through a diverse network of synaptic connections. As a consequence, ACs display a rich variety in terms of both their function and morphology, and 30–40 different types have already been identified till the present day [90,91,92]. A seminal study by Haverkamp and Wässle on CaBPs in the mouse retina [36] was among the earliest works that provided valuable insight into the expression of CaR, CaB and PV (among others) in mouse ACs. They found that CaR was the most intensively expressed marker, labelling a large number of regularly located (somata in the INL) and displaced (somata in the GCL) ACs (Figure 3). Due to the extensive CaR expression in a number of RGC populations (see below), the distinction of CaR-expressing RGC and displaced AC can be challenging for the experimenter. Haverkamp and Wässle showed that immunolabels to both CaR and CaB (but not PV) resulted in a distinct three-layered stratification whose inner and outer bands co-localized with the cholinergic starburst AC fibers in the IPL. Starburst cells have been identified in all examined mammalian species; they display a characteristic radial dendritic arbor and provide asymmetric GABAergic inputs to ON–OFF direction selective GCs (ON-OFF DS RGC), which is the foundation of the direction selective response [93,94,95,96]. Besides the two peripheral cholinergic bands in the IPL, the third band in the middle is provided by the WA-S2/3 wide-field AC population [97], which contribute to the populations of CaR-stained cells in both the INL and the GCL. While similar CaR and CaB labels have been detected for the rat and hamster retinas [45,98,99], starburst cells in non-murine mammalian retinas appear negative for a-CaR and a-CaB labels [39,40]. Instead, CaR immunoreactivity appears to be common in AII ACs in most examined species including the human retina as well (Figure 3) [39,40,99,100,101]. AII ACs have been shown to relay crossover inhibition for OFF pathways and they are also essential elements in the rod signal streams [102]. Interestingly, AII cells of the same mammalian species appear to maintain two CaBP systems as they also express PV [45,100,103]. However, the mouse retina seems to be an exception in this regard as mouse AII cells appear PV negative in immunolabels [104,105,106]. Does negative PV and CaR staining of mouse AII ACs in immunohistochemical approaches make these neurons different from their non-murine counterparts? Contrary to the negative CaR and PV immunonegativity, AII cells in the CaR-GFP mouse line display heavy GFP staining [107,108]. This suggests that CaR in AII cells is either present in a conformation that is not recognized by most commercially available antisera or that the CaR protein conformation in these cells changes during classical fixation protocols. Whether a similar labeling mismatch also occurs for PV in mouse AII cells requires further examination. However, the closely related rat maintains PV+ AII ACs [105,109], suggesting similar expression. Besides AII cells, PV is localized in the rat retina in both regularly placed and displaced wide-field ACs. Although there are PV- and PV/CaR-dual-stained ACs in the mouse retina [110], their identity remains unknown. This indicates that retinas of close relative species such as the mouse and the rat can display very different CaBP staining, indicating that the presence of these proteins is not cell type specific.

It is challenging to compare the above description on CaBP expression in the mouse retinal ACs (and other animal models) with those reported in humans [44] due to the dissimilar look and different nomenclature of human ACs [54]. The identity of many PV-, CaR- and CaB-stained human ACs remain unknown. However, some of them clearly belong to either stellate or semilunar ACs (Table 1). Semilunar and stellate cells maintain long, mostly radial running dendrites. Thus, they can be considered as the human counterparts of some wide-field ACs in other mammalian species, including the mouse. In addition, there are clear consistencies in CaBP expression of these species (and other mammals). For example, CaBPs can be co-expressed in the same ACs such as in the case of the CaR/CaB-dual-stained semilunar type 2 (or A19) ACs, some CaB/PV-expressing star-shaped middle-field ACs and unidentified CaR/PV-expressing ACs [44]. Furthermore, human AII cells express all the three major CaBPs (PV, CaR and CaB) and their intracellular localization seems compartment specific [44]. The presence of a further EF-hand CaBP, the SCGN was detected in human ACs as well. Among them, the type 1 tyrosine-hydroxylase-expressing cells (TH1) were identified as either SCGN+ or SCGN/CaR-dual-labeled [44]. TH1 ACs are polyaxonal cells (besides dendritic fibers they also maintain thin axonal processes; [111,112,113,114]) and have been identified in all examined mammalian retinas. They are involved in light-dependent dopamine release, by which they control the light adaptation of retinal neurons [115,116]. The co-expression of SCGN with other CaBPs was also observed within an SCGN/CaR/PV-triple-labeled human AC population whose identity remains unclear [44]. The case of human TH1 cells is also a good example for the compartment-specific expression of various CaBPs as neither CaR nor SCGN is present in the characteristic perisomatic rings TH1 processes form around other AC somata. In addition, the presence of SCGN in proximal dendrite segments is clearly more prominent than CaR. The expression of CaR was also reported by TH2 (but not in TH1) ACs in the rat retina. However, a similar CaR expression of mouse TH2 cells was not confirmed [110].

## 7. CaBP Expression in GCs

RGCs in the primate, monkey, cat, rabbit, and mouse retinas include a number of various morphological subtypes [117]. The classification scheme has been primarily based on morphological criteria, including the dendritic stratification within the inner plexiform layer (IPL), the extent of the dendritic field and the density of branching. Besides the most common small-field (midget in primates, beta in cats) and large-field RGCs (parasol in primates, alpha in cats, rabbits, rats and mice), retinas of all species contain a number of other subtypes; 15 in primates [118,119], at least nine in cats [120,121,122,123,124], 11 in rabbits [125] and 20–30 in mice [126,127,128,129,130]. Functional classification of mammalian RGCs has been performed via electrophysiology and/or Ca^2+^ imaging and the matching of morphological and functional populations has been a subject of ongoing research. The light-evoked RGC responses could be ON, OFF or ON/OFF based on their polarity, but there are great differences in response kinetics that could also serve as a basis for classification.

Like the model species, over 20 different GC subtypes of the human retina have been characterized based on morphological criteria by Kolb and colleagues [54]. M (magnocellular) and P (parvocellular—midget) cells appear to be the most numerous in the human retina, while the remaining subtypes are less represented and are located mostly in the extrafoveal retinal regions. Recent work by Kántor and colleagues [44] showed that at least a third of these morphologically identified cells express one of the main three discussed CaPBs (3 CaR+, 1 CaB+, 1 PV+, 1 CaR/CaB+, 1 CaR/PV+ and 1 CaB/PV+ RGC; Table 1). However, the immunocytochemistry approach utilized in that study did not allow for the identification of most of these RGC populations. When the antibody labels were successfully combined with Lucifer Yellow dye injections, it became clear that the PV+ RGCs display soma-dendritic morphological features of M cells. On the other hand, it remains unclear if the small receptive field P cells that are responsible for contrast detection express any CaBPs in the human retina. The high portion of CaBP-expressing RGC subtypes of the human retina ([46]; Kántor colleagues [44]) is in accordance with similar findings with most of the examined monkey species [43,131]. In addition to RGCs, retino-recipient P and M LGN layers are rich in CaBP-positive RGC axonal terminals in the adult monkey as well [132]. This is in agreement with the PV immunopositivity of parasol (or M) cells that project to the M LGN layers.

The CaBP expression patterns of RGCs in the mouse retina, as one of the most popular models, appear somewhat similar to the human retina (Figure 3). One of the similarities is the expression of PV in mouse OFF alpha RGCs ([68]; called PV5 cells by the authors) that is likely the mouse homologue of the primate parasol cells. OFF alpha cells also express CaR in mice [107] as a secondary CaBP (Figure 3, Table 1). Although PV/CaR co-expression was not confirmed for human parasol cells and one unidentified RGC population appears dual-labeled in the human specimen as well, which (based on purely soma size) could be a parasol cell [44]. These data suggest that, at least in the case of the parasol/OFF alpha RGC system, the CaBP signature is consistent for these two species (Figure 3, Table 1). Recent single cell injection and patch-clamp studies have revealed that, besides OFF alpha cells, PV is expressed in many mouse RGC subtypes [68,133,134]. Among the well-known subtypes, the intrinsic photoreceptive RGCs (ipRGCs) appeared negative for PV [135], whereas the ON–OFF direction selective (PV2), two large field (PV1 and PV6) and three small/medium-sized RGCs (PV3, PV4 and PV7) were GFP+ in the retina of the PV-GFP mouse [68]. The heterogenous nature of PV-expressing GCs has been shown by immunocytochemical means as well: eight different morphological types of mouse GCs were found PV+ (A1, B3, B4, C1, C2, C4, C5, and D2 [133]; nomenclature was adopted from [126]). The difference in nomenclature of the PV-GFP and the immunocytochemistry studies might be confusing. However, it can be concluded regardless that PV is abundantly expressed in mouse RGCs. Considering a similar abundance of PV+ RGCs in the human retina [44], it is possible that, besides the above-mentioned parasol/OFF alpha system, further overlap in PV expression exists between human and mouse RGC homologs.

Following dye injections into CaR-immunolabeled mouse RGCs, Lee and colleagues identified 10 subtypes ([136]; A2, B1, B2, B3, B4, C2, C3, C4, C5 and D2; nomenclature was adopted from [126]). Among these CaR+ RGCs, eight subtypes (B1, B2, B3, B4, C2, C4, C5 and D2) with small/medium dendritic fields sent axon projections to the colliculus superior [137]. CaB, the third among the presently discussed CaBPs, is also expressed by at least 10 mouse RGCs ([138]; A2, B1, B2, B3, B4, C2, C3, C4, C5 and D2; the nomenclature was adopted from [126]). These data together with the above PV and CaR labels indicate that A1 and C1 cells express only PV, whereas CaB/CaR dual expression is present in four RGC populations (A2, B1, B2 and C3), CaB/CaR/PV triple expression in five populations (B3, B4, C2, C4, C5 and D2) and two populations (C6 and D1) are not expressing any of these three CaBPs [133,136,137].

Based on the above descriptions, one may conclude that most mouse RGCs express two or three CaBPs simultaneously. While this might be the case for some RGCs, it may not be a general phenomenon. Authors of the dye injection studies note that approximately one-third of the ON–OFF DS RGCs expressed CaR, one-tenth expressed PV and one-third of ON DS cells were PV+ [136]. According to this note, CaR and PV are not expressed by all cells in a population but only in a portion of the cells, which calls the validity of CaBPs as cell-specific markers of mouse RGCs into question.

It is unknown how the presence of each CaBP modifies the spiking characteristics of RGCs and thereby how the final visual code is affected. The best-known PV+ neurons in most brain areas are the fast-spiking, GABAergic, inhibitory interneurons (e.g., basket cells; reviewed by [139]), whose response characteristics may partially be due to the expressed PV (see above). Whether PV has similar effects on light-evoked responses in human and mouse RGCs (e.g., parasol and OFF alpha RGCs) is not yet known. Human parasol and mouse OFF alpha cells are typically considered to belong to the transient spiking cells, which seems to be in contradiction with this hypothesis. However, OFF alpha cells (PV5 or A2 in [68] and [133]) are also capable of maintained spiking if the adequate approach signal is presented [68].

Further experiments are required to prove whether the human counterparts of the CaR-, PV- and CaB-expressing mouse RGCs exist, and whether their CaBP signature is similar to those exhibited by mouse RGCs. Until then, it remains uncertain whether the mouse retina serves a good model for human RGC studies.

## 8. CaBP Expressional Changes in Retinal Neurons

In the previous sections, we provided an extensive overview on cell types of the mammalian retina (with focus on the mouse and human retinas) regarding the expression of the three discussed CaBPs, which have generally been utilized as cell type-specific markers. However, it has been shown that CaBP expression can change under certain neurodegenerative conditions. For example, a reduced PV and or CaR expression was reported in cells of the INL and GCL as a result of diabetic retinopathy, retinal ischemia and glaucoma [79,109,140,141,142]). PV is expressed mostly by cells with glutamatergic inputs, so it might be possible that PV could have a protective effect against Ca^2+^ imbalance. Due to the changing PV expression levels in several neurodegenerative conditions, it has been suggested that PV also plays a role in determining cell survival [143,144,145,146,147,148]. However, only the downregulation of expression and not a selective survival of PV-expressing RGCs and displaced ACs was detected following optic nerve crush [149]. Besides PV, CaB and CaR may also play a neuroprotective role against glutamate toxicity [150,151,152]. It has been suggested that high levels of CaB are neuroprotective in HCs. In a rat model of retinal ischemia, most PV+ and CaR+ retinal cells were shown to succumb to ischemia and reperfusion injury, whereas CaB-expressing HCs [79] and CaB+ cells in the GCL seemed more protected against injury [153]. It has been shown that a CaB overexpression reduces excitotoxic cell death in other CNS areas as well [154,155]. All this evidence converges to the conclusion that, at least under pathological conditions, CaBP expression can change dynamically. Besides pathologically induced CaBP expressional changes, PV expression in the rat retina has been reported to show dynamic circadian rhythm mediated changes [156] as well. In this case, PV expression levels in AII cells changed by 15-fold during daily cycles and additional lightly labeled PV+ neurons (BC and HC) emerged in certain periods (ZT 14, 16 h [17,19]).

## 9. Concluding Remarks on CaBP Expression in Retinal Neurons

This review summarizes published results on the expression of CaBPs in mammalian retinas. One of the main purposes of this summary was to see whether CaBPs can serve as type-specific markers of retinal neurons for experimentation. In certain cases, CaBPs are expressed consistently by members of a single (or a couple of) cell type, such as in the case of REC for type 2 mouse BCs and human DB2 cells. However, this appears to be a rare case and the rest of the CaBPs included in this study, particularly the ones in focus of this review (PV, CaB and CaR), are expressed by heterogenous neuron populations in both the mouse and the human retinas (and all other mammalian species as well). In these latter cases, the CaBP-expressing outer retinal neurons (PR, HC and BC) have been identified morphologically as well (see the above descriptions). However, in the case of the mouse and human inner retinal neurons, the picture seems to be more complex. While certain cell types have been identified, including the CaB/CaR-expressing mouse starburst cells and the CaR/CaB/PV-expressing AII cells in the human retina, the identity of many other CaBP+ is unknown in both species. It is obvious that further experimentation is needed to fill the remaining gaps.

At the same time, the identities of PV+ mouse ACs and CaR-, CaB- and PV-expressing human GCs are poorly studied. Once a comprehensive characterization scheme becomes available for both species, CaBPs may prove to be suitable as type-specific markers for inner retinal neurons as well. However, the heterogeneity of CaBP-labeled populations necessitates the application of a second (or third) neurochemical marker and/or the study of certain morphological characteristics as well to sort the labeled populations into groups of distinct types and possibly subtypes. There are two additional caveats concerning the issue of CaBPs as specific neuronal markers in the retina, with the first one being the reliability of the applied markers and by extension, the recorded signal. As we outlined above, circadian rhythmicity, for example, can alter PV expression in the rat retina [156] and thus, similar expression-altering conditions may exist for the other two CaBPs in mammals as well. In addition, CaBP expression may also change considerably under pathological conditions [79,109,140,141,142]. These few examples thus suggest that CaBP expression in retinal neurons is not static but rather dynamic, by which neurons adapt to their constantly changing environment, including tissue stress, pathological insults and normal daily physiological rhythmicity. These findings challenge the view of CaBPs as cell type-specific markers, necessitating a switch in perspective for their role as function- and/or condition-specific markers for certain cell populations instead.

The second aim of this review was to see whether the expression of CaBPs determines the excitability and/or activity patterns of retinal neurons. These physiological parameters have often been measured by examining their light stimulus-evoked membrane potential changes and action potentials possibly generated upon this activation. As discussed above, the different kinetics of Ca^2+^ binding by PV and CaB/CaR may modify the response kinetics by altering the duration of different phases of the evoked intracellular Ca^2+^ increments. In this scenario, the presence of intracellular PV should buffer later phases (> 100 ms) of Ca^2+^ waves, thereby making neuronal responses more transient. On the other hand, CaB and CaR (being relative fast Ca^2+^ buffers) chelate intracellular Ca^2+^ from the beginning of light-evoked Ca^2+^ waves, meaning that the transient response component would be truncated as well, which would eventually lead to more sustained responses in turn. CaB has also been shown to interfere with the Ca^2+^-dependent inactivation of Ca^2+^ currents as well as further slowing down the response decay, thus prolonging light-evoked responses. Whether this functional aspect is relevant for retinal neurons or not has not been explored in depth. In certain cases, such as PV+ mouse alpha GCs and human parasol cells, the hypothesis might seem relevant as both GC populations tend to respond with transient spiking to light flashes. Moreover, PV seems to be a more specific marker for inner retinal neurons, where AC and GC light responses tend to be more transient than the neurons of the outer retina. HCs, for example typically provide sustained responses and they rarely express PV in mammals. On the other hand, CaB is typically expressed by many outer retinal cells, such as PRs, HCs and BCs (in the human and other mammalian species but not in the mouse), that very often maintain sustained light responses. Sustained neurons exist among inner retinal ACs and GCs as well, and so do CaB- and/or CaR-expressing cells. Therefore, this functional expression of CaBPs that determines neuronal response kinetics might as well be a line of research worth pursuing in the mammalian retina.

The third and last aim of this review was to compare CaBP expression in the mouse and human retinas to determine the applicability of collected mouse data for future studies on the human retina. In this regard, one can conclude that there are major differences between the two species (Figure 3, Table 1). These, among many others, include: (i) no CaBP is expressed by mouse PRs but human cones are CaB+; (ii) no CaBPs are expressed by mouse BCs, while several human BC subtypes express CaB, CaR and PV; (iii) starburst cells are CaR/CaB-dual-labeled in the mouse but not in the human retina; (iv) AII cells are CaR/CaB/PV-triple-labeled in the human retina, while mouse AII ACs could be visualized in the CaR-GFP transgenic lines but appear unstained in any CaBP immunolabels, including those testing for the expression of CaR. Nevertheless, before condemning the mouse retina as an inadequate model for human-oriented studies, it is important to draw attention to the few observed similarities: AII cells of both mice and humans express CaR (as demonstrated in CaR-GFP lines in the mouse retina) and the presence of PV has been established in both the mouse alpha and the human parasol GCs. However, the identity of most CaBP-expressing human GCs and ACs remains unknown and PV-expressing mouse ACs have not been characterized morphologically either. Therefore, one might expect that at least in the case of inner retinal neurons, future experimentation should reveal further similarities and overlaps between retinas of the two species. Until then, data collected in the mouse as a model can still be used with confidence in the few aforementioned cases, but only with caution regarding other neuronal populations.

## Figures and Tables

**Table 1:** Cell types expressing major EF-hand CaBPs (CaB, CaR, PV) in the mouse and human retina.

	Calbindin					Calretinin					Parvalbumin				
	PR	HC	BC	AC	GC	PR	HC	BC	AC	GC	PR	HC	BC	AC	GC
**Mouse**		1. **B type**		1. Stb-a 2. Stb-b 3. WA type 2/3	1. A2 2. B1 3. B2 4. B3 5. B4 6. C2 7. C3 8. C4 9. C5 10. D2				1. Stb-a 2. Stb-b 3. WAtype 2/3 4. **AII**	1. PV5/Off alpha/A2 3. B1 3. B2 4. B3 5. B4 6. C2 7. C3 8. C4 9. C5 10. D2/ON-OFF DS		1. **B type (?)**		1. UI	1. PV1 2. PV2/ON-OFF DS/D2 3. PV3 4. PV4 5. **PV-5/Off alpha** 6. PV6 7. PV7 8. A1 9. B3 10. B4 11. C1 12. C2 13. C4 14. C5
**Human**	1. S 2. L/M	1. **HII**	1. DB3 2. DB4 3. DB5 4. DB6 5. IMB (?)	1. Semilunar 2. Stellate 3. AII 4. UI	1. UI			1. IMB or DB4	1. Semi-lunar 2. Stellate 3. type1 TH AC 4. Star-shaped middle-field AC 5. **AII** 6. UI	1. bistratified 2. UI		1. **HI** 2. **HII**	1. giant diffuse or giant bistrati-fied	1. star-shaped 2. middle-field 3. AII 4. UI	**1. parasol** 2. UI

Marked as underlined and bolded if the same cell type is labeled in both species; UI: unidentified cell type.

## Abbreviations

AC	amacrine cell
BB	blue cone-specific bipolar cell
BC	bipolar cell
CaB	calbindin
CaBP	Ca^2+^-binding protein
CaM	calmodulin
CaMKII	calmodulin kinase II
CaR	calretinin
CNS	central nervous system
dAC	displaced amacrine cell
DB	diffuse bipolar
DS	direction selective
FMB	flat midget bipolar
GABA	gamma-Aminobutyric acid
GC	ganglion cell
GCL	ganglion cell layer
GFP	green fluorescent protein
HC	horizontal cell
IMB	invaginating midget bipolar
INL	inner nuclear layer
IPL	inner plexiform layer
L (PR)	long
LGN	lateral geniculate nucleus
LTP	long-term potentiation
M (GC)	magnocellular
M (PR)	medium
ONL	outer nuclear layer
OPL	outer plexiform layer
P	parvocellular
PKC	protein kinase C
PR	photoreceptor
PV	parvalbumin
REC	recoverin
RGC	retinal ganglion cell
S (PR)	short
SCGN	secretagogin
SCN	suprachiasmatic nucleus
Stb	starburst
TH	tyrosine-hydroxilase
UI	unidentified
WA	wild field amacrine
ZT	zeitgeber time
